# *YAP1* acts as oncogenic target of 11q22 amplification in multiple cancer subtypes

**DOI:** 10.18632/oncotarget.1844

**Published:** 2014-03-23

**Authors:** Erica Lorenzetto, Monica Brenca, Mattia Boeri, Carla Verri, Elena Piccinin, Patrizia Gasparini, Federica Facchinetti, Sabrina Rossi, Giuliana Salvatore, Maura Massimino, Gabriella Sozzi, Roberta Maestro, Piergiorgio Modena

**Affiliations:** ^1^ Experimental Oncology 1, Aviano National Cancer Institute, Aviano (PN), Italy; ^2^ Tumor Genomics Laboratory, Milan National Cancer Institute, Milan, Italy; ^3^ Department of Pathology, Treviso General Hospital, Treviso, Italy; ^4^ Department of Biology and Cellular and Molecular Pathology, University Federico II, Naples, Italy; ^5^ Unit of Pediatric Oncology, Milan National Cancer Institute, Milan, Italy; ^6^ Laboratory of Human Genetics, Sant' Anna General Hospital, San Fermo della Battaglia (Como), Italy

**Keywords:** YAP1, Gene amplification, Salvador-Warts-Hippo pathway, Lung cancer, Cervical cancer, Oncogene

## Abstract

The transcriptional coactivator *YAP1* is a critical effector of the human Salvador-Warts-Hippo pathway. Literature data report apparently discrepant results on the carcinogenic role of *YAP1*, which acts either as oncogene or as tumor suppressor in different in vitro and in vivo models. Furthermore, genomic amplification events of 11q22 locus encompassing *YAP1* gene have been detected in multiple tumor types but there is limited direct evidence about the oncogenic role of endogenous *YAP1* within in the amplicon.

We screened a panel of human tumor samples and cancer cell lines and identified that the *YAP1* amplification event is actually present in up to 23% of the cases. We exploited EKVX (lung cancer), CaSki (cervical cancer) and RO82 (thyroid cancer) cell lines harboring both genomic *YAP1* amplification and *YAP1* protein overexpression, in order to study the effects of downregulation of endogenous *YAP1* by RNA-interference strategies. Class comparison analysis of gene expression profiling data identified 707 statistically significantly modulated genes (multivariable global test p-value = 0.002) that were functionally annotated for cell proliferation and cellular movement ontologies. Mechanistic studies of the identified perturbed pathways revealed that *YAP1* silencing significantly decreased cell proliferation and cell cycle perturbation associated with upregulation of p21 and p27 cell-cycle inhibitors, reduced cell migration (p<0.048) and anchorage-independent growth (p<0.02). In CaSki cell line, *YAP1* silencing induced significantly increased sensitivity and cell-death response to cisplatin treatment (p=0.011) as well as reduction of in-vivo tumorigenic potential (p=0.027).

Overall, these results establish that *YAP1* is a direct oncogenic target of the 11q22 amplicon in previously unreported cancer types and support the relevance of such genetic aberration in carcinogenesis in a fraction of multiple tumor types.

## INTRODUCTION

The recently described Salvador-Warts-Hippo pathway (SWH or Hippo pathway) is highly conserved throughout evolution [1] and is crucially involved in organ size regulation [2,3]. Hippo pathway consists of several negative growth regulators acting in a kinase cascade that ultimately inactivates Yorkie in *Drosophila* or *YAP1* (Yes-associated protein 1) in mammalian; these two effector proteins are both transcriptional coactivators that positively regulate cell growth, survival and proliferation [4]. Therefore *YAP1*, which is localized in 11q22 genomic region, represents a critical downstream regulatory target of this signaling pathway [5].

Literature data so far described the involvement of *YAP1* in carcinogenesis contradictorily. Initially, *YAP1* was classified as a tumor suppressor gene (or at least as helper of tumor suppressors), as it was reported to exert pro-apoptotic functions. Following DNA damage, *YAP1* functions as a co-activator of TP73-mediated apoptosis in TP53 null cells [6, 7], after phosphorylation of *YAP1* at tyrosine 357 [8], following *YAP1* dissociation from cytoplasmatic multiprotein complex with 14-3-3 and Akt [9] and as well as a result of RASSF1A activation [10]. As a consequence *YAP1* translocates into the nucleus promoting the assembly of the active complex inducing the transcription of target genes [7]. *YAP1* was also proposed to be a tumor suppressor in breast cancer, as the target of loss of heterzygosity in 11q22 genomic region [11].

On the contrary, *YAP1* was also described to function as an oncogene by promoting increased organ size and cancer development. *YAP1* resulted amplified in human hepatocellular carcinoma and cooperated with *myc* oncogene to induce tumor growth in nude mice [12].** In non-transformed mammary cells *YAP1* ectopic overexpression induces alterations typical of a transformed phenotypes, namely anchorage-independent growth, EMT, growth factor independent proliferation, activation of AKT/ERK and inhibition of apoptosis [13]. In addition, in transgenic mouse models the liver-specific *YAP1* overexpession induced a dramatic increase of liver organ size, eventually leading to cancer development [14, 15]. Moreover, recent data indicated that *YAP1* activity correlates with high histological grade and metastasis in breast cancer [16]. Furthermore, the 11q22 genomic region was found amplified in individual cases of several human tumor types [12, 17-29] but the direct evidence of *YAP1* amplification is described in very few of these cases [12, 21, 24, 26-28].

Notably, *YAP1* point/small mutations have not been described so far and the reported 11q22 amplification events include multiple flanking genes in addition to *YAP1*. Some of these genes have been involved in cancer development, including a cluster of matrix metalloproteinase (*MMP*) genes [30], two members of the BIRC family of caspase inhibitors (*BIRC2* and *BIRC3*) [31] and the progesterone receptor (*PGR*) [32]. As a consequence, there is no direct evidence about the oncogenic role of endogenous *YAP1* in the context of cancer cells carrying the 11q22 amplification event.

In the present work we corroborate that *YAP1* plays an important role in the tumorigenic phenotype of 11q22-amplified cancer cell lines, as it effectively supports multiple transformed properties. Moreover we detect *YAP1* copy number amplification in clinical series of different human tumor types and identify the downstream genes and pathways that are critical as *YAP1* effectors in carcinogenesis.

## RESULTS

### Identification of cancer cell lines and clinical specimen carrying 11q22 amplification and *YAP1* overexpression

Public and private genomic copy-number databases were interrogated for the copy number status of loci encompassing *YAP1* (Supplemental Table 1). Notably, homozygous deletion encompassing *YAP1* gene was a very rare event, in fact it was found only in 3/664 (0.5%) cancer cell lines and in 3/1629 (0.2%) cancer tissue samples. In contrast, *YAP1* copy amplification event was found in a greater percentage of the same samples (Chi-square test p<0.0001). In fact, it was reported in 40/664 (6%) cancer cell lines, in 31/1629 (1.9%) cancer tissue samples, in 2/110 (1.8%) primary cancer cell cultures and in 1/20 (5%) xenograft tumors (Supplemental Table 1).

**Figure 1:**
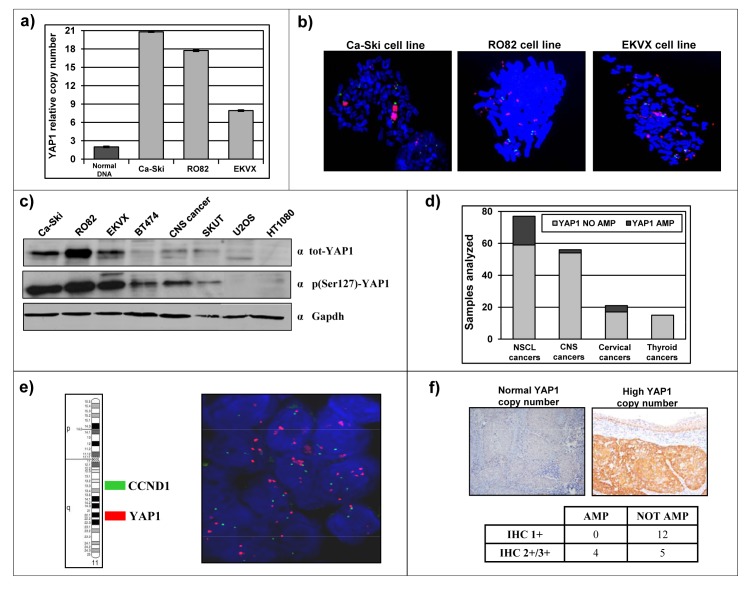
Identification of cancer cell lines and clinical cancer samples carrying 11q22 amplification and overexpression a) Gene dosage analysis by qPCR shows *YAP1* copy number amplification in Ca-Ski, RO82 and EKVX cell lines (grey bars). Normal DNA (2 copies of *YAP1*) was used as a control (black bar). b) FISH analysis defined the presence of the amplicon spanning *YAP1* gene in Ca-Ski, RO82 and EKVX cell lines (Green = Control BAC clone; Red = *YAP1*-containing BAC clone). c) Total and phospho (Ser127)-*YAP1* protein level in Ca-Ski, RO82 and EKVX cell lines (high protein levels) and in other cancer cell lines. GAPDH was used as loading control. d) Number of cases with normal *YAP1* copy number (in grey) or *YAP1* copy number amplification (in black) in NSCL non small cell lung cancers samples, CNS central nervous system cancers samples, cervical cancers samples and thyroid cancers samples. e) Representative FISH analysis of a NSCLC sample carrying *YAP1* amplification and normal CCND1 (located in 11q13) copy number. (Green = CCND1-containing BAC clone; Red = *YAP1*-containing BAC clone). f) Representative immunohistochemistry analysis of *YAP1* protein level in a cervical cancer sample with normal *YAP1* copy number (left panel) and a sample carrying *YAP1* copy amplification (right panel). Fisher's exact test two-tailed p=0.0211.

We focused our attention on tumor subtypes with little or no established involvement of *YAP1* gene, and selected representative cancer cell lines, including Ca-Ski cell line (Cervical squamous cell carcinoma), RO82 cell line (Follicular thyroid carcinoma) and EKVX cell line (Non small-cell lung adenocarcinoma). Preliminary experiments were performed in order to verify *YAP1* amplification in these established cancer cell lines and to evaluate the occurrence of *YAP1* protein overexpression. Ca-Ski, RO82 and EKVX cell lines showed *YAP1* high-copy number (Figure 1a). FISH analysis allowed to define that *YAP1* copy amplification is contained in a homogeneously staining region in Ca-Ski cell line or as multiple interspersed copies (double minutes) in RO82 and in EKVX cell lines (Figure 1b). *YAP1* amplification correlated with protein overexpression as detected by western blot analysis. In fact, Ca-Ski, RO82 and EKVX cell lines show high protein level of both total- and phospho(S127)- *YAP1*, compared to other cancer cell lines lacking 11q22 amplification (Figure 1c). Mutational analysis by exon amplification and sequencing was performed, but no small/point mutations were detected in Ca-Ski, RO82 and EKVX cancer cell lines (not shown). The major known genetic and epigenetic traits of the cell lines under study is reported in Supplemental Table2.

Literature data report that chromosome region 11q22, containing *YAP1* gene, is amplified in a small percentage of samples from multiple human tumor types [12, 17-29]. We directly investigated the frequency of *YAP1* copy number amplification in clinical series from different human tumor types and revealed *YAP1* amplification in 4/25 (16%) cervical cancer samples, in 18/77 (23%) non small cell lung cancers (NSCLC), in 2/56 (3.6%), central nervous system tumors (CNS) and in 0/15 thyroid cancers (Figure 1d). The 77 NSCLC samples arrayed on a tissue microarray were assayed using double-colour FISH. Since cyclin D1 is present in the same chromosomal arm as *YAP1* but in 11q13 and CCND1 is frequently amplified in lung cancer [33, 34], we used one probe specific for cyclin D1 gene and another probe specific for *YAP1* gene or FISH analyses. Using such approach, we detected a large amplification which included both *YAP1* and CCND1 genes in 16/77 (21%) NSCLC samples, while 2/77 samples (2.6%) carried an amplification restricted to *YAP1* (Figure 1e). We investigated the *YAP1* copy number status of the other clinical samples, which were available as individual paraffin blocks, by a quantitative, real-time PCR approach. *YAP1* amplification was found in 4/25 (16%) cervical cancer samples, including high copy amplification in 3/4 and low copy amplification in 1/4. *YAP1* protein expression was assayed by immunohistochemistry in these cervical cancer samples, revealing that *YAP1* protein level was higher in samples carrying *YAP1* copy amplification compared to samples with normal *YAP1* copy number (p=0.021, Figure 1f).

### *YAP1* is efficiently downregulated in 11q22-amplified cancer cell lines

**Figure 2:**
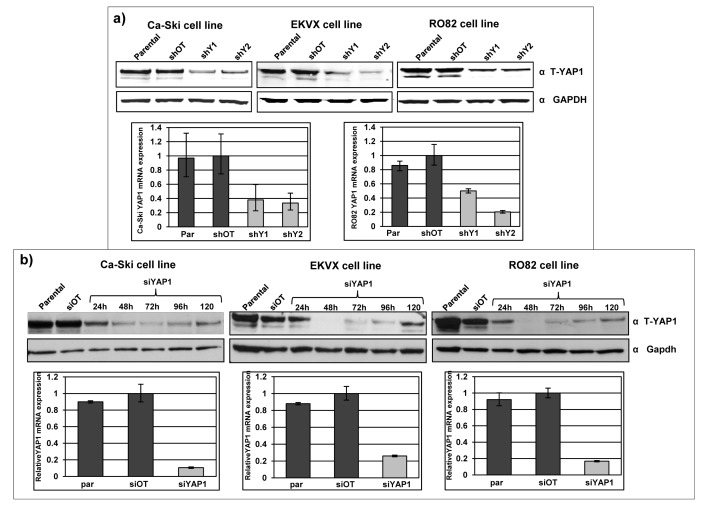
Effective *YAP1* silencing in 11q22-amplified cancer cell lines a) *YAP1* protein expression in Ca-Ski, EKVX and RO82 bulk cell population after puromycin selection of off target cells (shOT) and *YAP1* silenced cells (shY1 and shY2) was defined by western blot analysis using anti-total *YAP1* antibody. GAPDH was used as loading control. Lower panel: *YAP1* mRNA expression level in Ca-Ski and RO82 parental cells, off target cells (shOT) and *YAP1* silenced cells (shY1 and shY2). *YAP1* mRNA expression level was determined by real-time quantitative PCR (qPCR) analysis. b) *YAP1* protein level in Ca-Ski, EKVX and RO82 parental cells, off target cells (siOT) and *YAP1* silenced cells (si*YAP1*) was determined by western blot analysis after short-term, siRNA-mediated silencing. The panel shows a time-course analysis 24 to 120 hours after transfection with si*YAP1*. Lower panel: *YAP1* mRNA expression level in Ca-Ski, EKVX and RO82 parental cells, off target cells (siOT) and of *YAP1* silenced cells (si*YAP1*), 24 hours post transfection, as determined by qPCR analysis.

Endogenous *YAP1* expression was modulated in Ca-Ski, EKVX and RO82 cell lines by RNA interference strategies. *YAP1* silencing was performed using both short-hairpin RNA (shRNA) lentiviral particles and small-interfering RNAs (siRNA). In stably infected sh-*YAP1* bulk cell population, *YAP1* residual expression of mRNA was on average 30% and the *YAP1* residual protein expression was 15-30% (Figure 2a). In si-*YAP1* short-term silenced cells, *YAP1* mRNA was silenced with a residual expression of about 10-20% and the protein was efficiently downregulated with minimal expression between 48 and 96 hours (Figure 2b). The three individual siRNAs used effectively silenced *YAP1* as much as an equal total amount of the pool of the three combined siRNAs (Supplemental Figure 1a). *YAP1* protein level in parental and silenced cell lines was similarly quantified by multiple antibodies, raised against different *YAP1* epitopes (Supplemental Figure 1b).

### Identification of *YAP1* target genes in 11q22-amplified cancer cell lines

**Figure 3:**
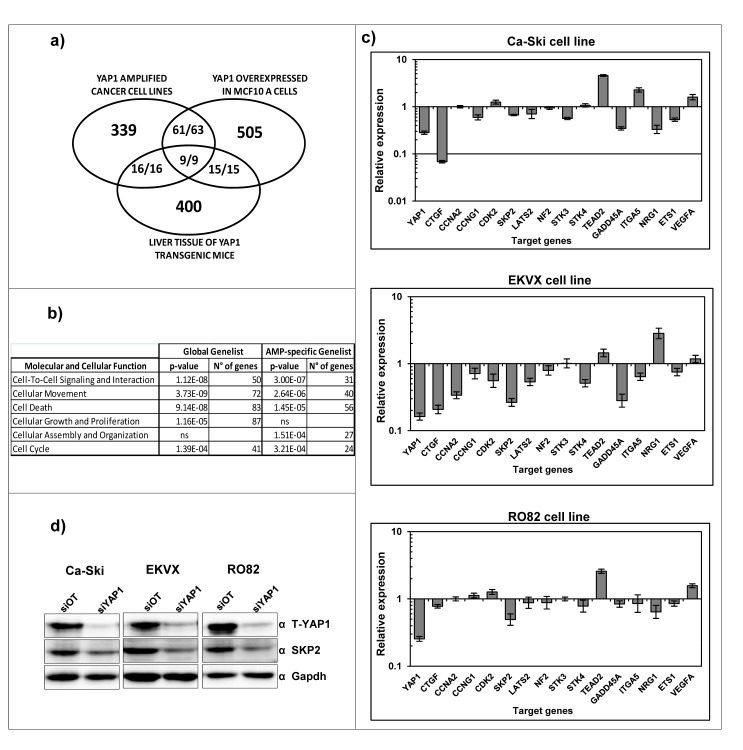
Identification of *YAP1* target genes in 11q22-amplified cancer cell lines a) Number of unique and common target genes identified in *YAP1* amplified cell lines following downmodulation of endogenous *YAP1* expression (Supplemental Table 2) and in normal cell contexts in which *YAP1* gene is overexpressed. The fraction numerators indicate the number of genes which are concordantly co-regulated in the studies. b) Molecular function pathways significantly modulated following downmodulation of endogenous *YAP1* expression. c) qPCR analysis showing the modulation of *YAP1* target genes in Ca-Ski, EKVX and RO82 cell lines after *YAP1* silencing using siRNAs. The siRNA off target was used as a calibrator. d) Total *YAP1* and SKP2 protein levels were analyzed by western blotting analysis in Ca-Ski siOT and si*YAP1* cells. GAPDH was used as loading control.

Literature data report that *YAP1* gene is a transcriptional cofactor [35] but *YAP1* target genes remain poorly characterized and therefore we performed gene expression profiling experiments with the aim to identify *YAP1* target genes that may mediate an oncogenic stimulus in *YAP1*-amplified cell lines. Using class comparison analyses comparing global gene expression profiles of *YAP1* proficient cells versus *YAP1*-silenced cells we identified 707 statistically significantly modulated genes at the uninominal p-value= 0.001 (multivariable global test p-value=0.002, Figure 3 and Supplemental Table 3). Among these target genes, 505 are down-regulated and 202 are up-regulated upon *YAP1* silencing (Chi-square p-value < 0.0001), thus suggesting that *YAP1* mainly acts as a transcriptional co-activator in the cancer cell lines under study. Using different tools of functional annotation, we found that the 707 genes identified are significantly enriched for gene ontologies related to cell proliferation and to cell movement molecular functions and suggesting that *YAP1* overexpression positively sustains these two biological functions (Figure 3b and Supplemental Table 3). In order to pinpoint the *YAP1* target genes specifically modulated in the context of *YAP1* amplification in cancer cells, we filtered our *YAP1*-gene signature with the *YAP1*-gene signature from two previous studies reporting gene expression profiling in normal cellular contexts in which *YAP1* gene is ectopically overexpressed [15, 36]. Among the 427 significantly regulated genes that could be compared across the three studies, we found that 88 are in common in our study and in at least one study from the literature and 86/88 are concordantly (up- or down-) co-regulated (9/9 genes concordantly regulated in all analyses) (Figure 3a). Indeed, these genes represent *YAP1* targets modulated in multiple different normal and tumor cell contexts. Additional 339 genes are uniquely modulated in the 11q22-amplified cancer cell lines (Figure 3a and Supplemental Table 3). We functionally annotated the filtered 339 gene-list using Ingenuity functional annotation tool, revealing that they were mainly annotated for cell proliferation and for cell movement ontologies again suggesting that *YAP1* overexpression positively sustains these two biological functions (Figure 3b and Supplemental Table 3), similar to the global 707 gene-list.

The validation of individual *YAP1* target genes identified by gene expression analysis was performed using qPCR on independent silencing experiments. We analyzed the modulation of CTGF gene [37], which represents one of the few known *YAP1* target genes, a group of genes involved in cell cycle regulation (CCNA2, CCNG1, CDK2, SKP2) [38-42]; the ITGA5 [43] and NRG1 genes involved in cell-cell interaction; the genes LATS2, NF2, STK3, STK4 and TEAD2, which are components of the Hippo pathway [44]; GADD45A gene which is involved in DNA repair [45]; VEGFA gene involved in angiogenesis [46] and ETS1 which is a transcription factor that acts as an oncogene via multiple pathways [47]. The genes modulated in agreement with the microarray data were 13 out of 16 in Ca-Ski and RO82 cell lines and 15 out of 16 in EKVX cell line (Figure 3c). Among them, the SKP2 protein was validated by western blotting on Ca-Ski, EKVX and RO82 cell lines revealing that protein levels are modulated in agreement with the microarray and qPCR results (Figure 3d).

### *YAP1* downregulation induces a moderate reduction of cell proliferation and influences the cell cycle

**Figure 4:**
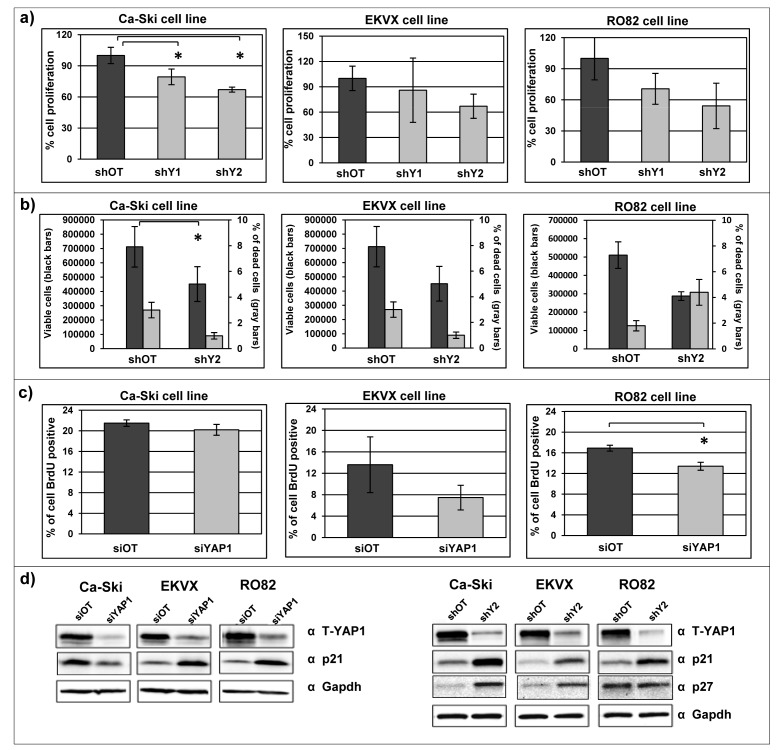
*YAP1* downregulation induces a moderate reduction of cell proliferation and perturbation of the cell cycle a) Sulforhodamine B assay was performed on Ca-Ski, EKVX and RO82 cell lines silenced for *YAP1* using stable silencing system. The percentage of cell proliferation was evaluated 72 hours post seeding. Means+ SEM from three independent experiments. In Ca-Ski cell line paired t-test shOT versus shY1 p=0.0124, shOT versus shY2 p=0.0157. b) Cell counting and trypan blue staining was performed on Ca-Ski, EKVX and RO82 cell lines silenced for *YAP1* using stable *YAP1* silencing system (shY2). The count of viable cells (left y axis) and the percentage of the dead cells (right y axis) were determined 72 hours after cells seeding. Means+ SEM from three independent experiments. In Ca-Ski cell line unpaired t-test shOT versus shY2 p=0.0079. c) BrdU assay was performed on Ca-Ski, EKVX and RO82 cell lines. The histograms showed the BrdU positive cells in siOT and in si*YAP1* cells. Mean percentage+ SEM from three independent experiments. In RO82 cell line paired t-test siOT versus si*YAP1* p=0.0215. d) p21 and p27 protein expression levels were analyzed by western blotting on Ca-Ski, EKVX and RO82 *YAP1* silencing cells (si*YAP1* and shY2) and off target cells (siOT and shOT). GAPDH was used as loading control.

Uncontrolled, aberrant cell proliferation is a common characteristic of aggressive cancer cells [48]. Therefore, in order to evaluate whether *YAP1* sustains the cell proliferation, as suggested by gene expression analyses, we investigated cell growth dynamics in 11q22-amplified cancer cell lines, using the bulk cell populations following sh-mediated stable silencing. As detected by both SRB assay and cell counting, *YAP1* silencing induced a moderate reduction in cell proliferation in all cell lines analyzed, which is statistically significant in Ca-Ski cell line (Figure 4a). Concordantly, *YAP1* silencing correlated with a reduction in viable cell counting without induction of cell death (Figure 4b). In addition, *YAP1* silencing induced a modest reduction of the proliferative cell compartment of cells in S phase of the cell cycle, as demonstrated by BrdU incorporation assay (Figure 4c). Notably, we detected a significant increase of the protein levels of p21 and p27, which are implicated in the negative regulation of cell-cycle [49, 50], in *YAP1* silenced cells compared to the off target cells in all *YAP1*-amplified cancer cell lines (Figure 4d).

### *YAP1* downregulation strongly reduces the tumorigenic phenotype

**Figure 5:**
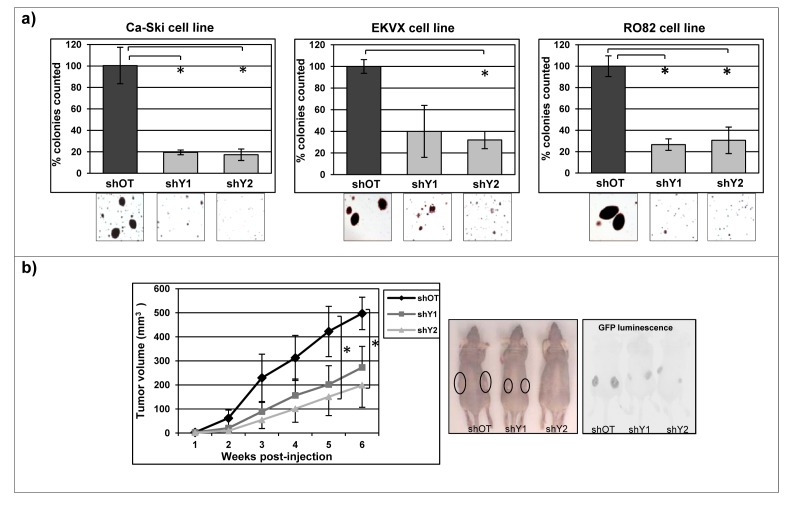
*YAP1* downregulation strongly reduces tumorigenic phenotype a) Soft agar assay was performed on Ca-Ski, EKVX and RO82 cell lines silenced for *YAP1* using stable sh-silencing system. Colonies were counted from 60 mm dishes and one randomly chosen field was photographed (10x magnification). The values correspond to the mean percentage + SEM from three independent experiments. In Ca-Ski cell line unpaired t-test shOT versus shY1 and shY2 p=0.009, in EKVX cell line unpaired t-test shOT versus shY2 p=0.022 and in RO82 cell line unpaired t-test shOT versus shY1 p=0.003, shOT versus shY2 p=0.012. b) *In vivo* mice tumor growth was analyzed on Ca-Ski cell line off target cells (shOT) and *YAP1* silenced cells (shY1 and shY2). The values correspond to the means + SEM from three independent experiments (12 mice per condition in total). Two-way ANOVA shOT versus shY1 and shY2 p=0.041 and p=0.027. A representative picture (left) and GFP luminescence (right) of tumors grown in mice 6 weeks after cells injection.

Normal cells typically are not able to grow and form cell colonies in semisolid media because under these conditions they undergo anoikis-mediated cell death. In contrast, aggressive cancer cells acquire the capability to grow without anchorage-dependence to the substrate, within a semi-solid medium such as agar [51]. In order to evaluate whether *YAP1* positively affected the anchorage-independent growth we evaluated the colonies growth in off target cells and in *YAP1* stably silenced cells. *YAP1* silencing induced a strong and significant reduction in the number and size of colonies counted in all 11q22-amplified cancer cell lines analyzed, compared to the control cells (Figure 5a). Furthermore, subcutaneous injection of stably infected Ca-Ski sh-off target and Ca-Ski sh-*YAP1* cells into nude mice showed a significant reduction in tumor volume in *YAP1* silencing cells, providing a direct evidence that reduction of anchorage-independent growth correlated with significantly reduced *in vivo* cell expansion of cancer cells (Figure 5b).

### *YAP1* silencing affects cell migration in Ca-Ski and in RO82 cell lines

**Figure 6:**
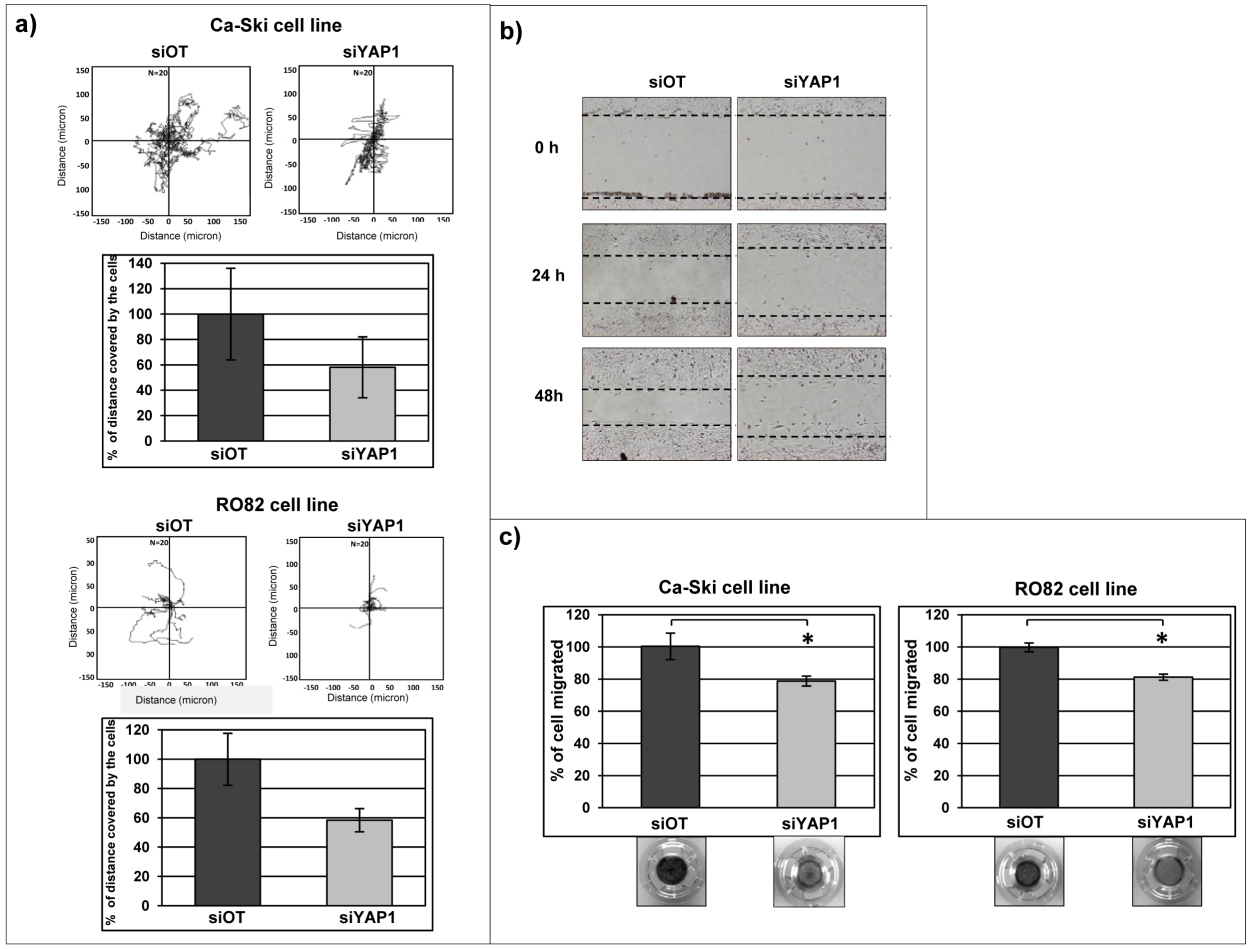
*YAP1* silencing affects cell migration in Ca-Ski and in RO82 cell lines a) Tracking experiment using time-lapse microscopy was performed on Ca-Ski and RO82 cells transfected with siRNA. The graphs show the distance covered by cells (micron) in 12 hours. The values in the histograms correspond to the percentage calculate on the means + SEM from three independent experiments. b) Wound healing assay was performed on Ca-Ski and RO82 cell lines depleted for YAP1 using siRNA silencing systems. The pictures show representative results. The T0 represents the cells 24 hours post-transfection (10x magnification). b) Chemotaxis experiment was performed on Ca-Ski and RO82 siOT and siYAP1 cells. The pictures show the cells migrated in low chamber of transwell stained with crystal violet. The values correspond to the means from one representative experiment out of three. In Ca-Ski cell line paired t-test siOT versus siYAP1 p=0.0487, in RO82 cell line paired t-test siOT versus siYAP1 p=0.0287.

Active migration of tumor cells is a prerequisite for tumor cell invasion and for metastasis development [52] and our gene expression analyses indicated that *YAP1* modulates several target genes that are reported to control cell-cell contact and cell migration. To investigate if migration activity is effectively affected by the modulation of *YAP1* expression, cell tracking analysis experiments using video time-lapse microscopy were performed in Ca-Ski and RO82 cell lines, while EKVX parental cell line was uninformative as it is characterized by very limited migration ability (not shown). In Ca-Ski and RO82 cell lines the short-term *YAP1* downregulation produced a moderate reduction of cell migration as measured by the distance covered by cells during 12 hours of observation, compared to the control off-target cells (Figure 6a). The phenomenon was qualitatively evident in RO82 cell line also by wound healing assay (Figure 6b), further supporting that the cell motility is reduced in *YAP1* silenced cells. The same results were obtained using either siRNA (Figure 6a-b) or shRNA (data not shown) cells. Similarly, cell migration was reduced after short term *YAP1* silencing in chemotaxis assay (Figure 6c).

### *YAP1* silencing increases DNA damage response in Ca-Ski cell line

**Figure 7:**
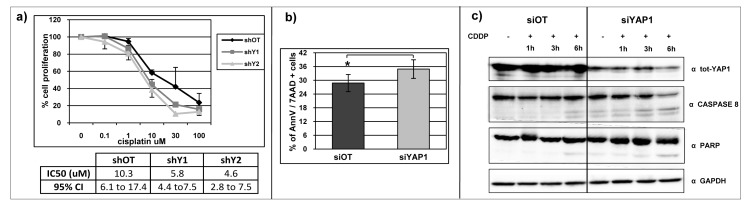
*YAP1* silencing increases DNA damage response in Ca-Ski cell line a) Percentage of cell proliferation reduction determined by SRB assay in Ca-Ski off target and *YAP1*-silenced cells after cisplatin treatment. The table shows the values of IC50 and the 95% confidence interval. b) Flow cytometer analysis was performed on Ca-Ski off target cells and *YAP1* silenced cells with siRNA silencing cell system. The histograms showed the the mean + SEM percentage of single or double annexin V and 7AAD positivity cells after cisplatin treatment. Paired t-test siOT versus si*YAP1* p=0.0107. c) Total *YAP1*, caspase 8 and PARP protein levels were analyzed by western blotting analysis in Ca-Ski siOT and si*YAP1* cells 1, 3 and 6 hours after treatment with 100 uM cisplatin. GAPDH was used as loading control.

In Ca-Ski cell line, *YAP1*-silenced cells are more sensitive to genotoxic stress induced by cisplatin treatment, compared to control cells (Figure 7a). We detected a statistically significant increase in annexin V and 7AAD positivity in *YAP1* silenced cells compared to the off target cells, indicating that in these cells the DNA damage induced a more prominent reduction of viability and increased cell death (Figure 7b). Concordantly, following DNA damage, we detected an increase in caspase 8 and PARP protein activation, which are essential mediators of the apoptosis process, in Ca-Ski *YAP1* silenced cells compared to the control cells (Figure 7c). To a lesser extent, cisplatin induced an analogous effect also in RO82 and EKVX cell lines (Supplemental Figure 2).

## DISCUSSION

In this study we demonstrate that *YAP1* effectively supports multiple transformed properties in 11q22-amplified cancer cell lines. In particular, in our experiments, we exploited RNA interference strategies to silence *YAP1* in cancer cell lines (Ca-Ski, EKVX and RO82) that carried 11q22 copy number amplification and *YAP1* overexpression and compared *YAP1* silenced cells and control cells for gene expression profiling, cell proliferation tumorigenic potential, cell migration and response to genotoxic stress.

Literature data report apparently conflicting results on *YAP1* role in carcinogenesis. In fact, *YAP1* has been reported to exhibit both oncogenic properties [12-15] and tumor-suppressive functions [7, 9, 10] in distinct *in vitro* or *in vivo* models. Our results provide definitive evidence that endogenous *YAP1* expression has oncogenic properties in cancer cells carrying *YAP1* gene amplification.

Several studies reported that the 11q22 locus is amplified in human tumor samples [12, 17-29]. The majority of the data published until now detected 11q22 amplification in different tumor types without directly investigating the *YAP1* copy alteration. We here provided direct evidence of *YAP1* copy amplification in up to 23% of samples from multiple tumor types, providing compelling evidence that it represents a cancer associated alteration in a fraction of multiple tumor types. In particular, we detected *YAP1* copy amplification in 4/25 (16%) of cervical cancer samples. Literature data reported 11q22 copy number amplification in cervical cancer samples [19, 25, 29], predominantly in patients with advanced stage of the disease [25]. These evidences, together with our data directly addressing *YAP1* copy amplification, support that *YAP1* is the target of the amplification at 11q22 locus in cervical carcinoma. Whether *YAP1* copy amplification event represents a marker of cervical cancer progression and prognosis remains an intriguing but unexplored issue.

Genes mapping within amplicons are considered as candidate oncogenes. Amplification is an important mechanism for gene copy number gain, for protein overexpression and sustained oncogene activation in tumor cells. Most amplification events include a relatively large region of DNA that contains several genes that can be considered possible targets of the amplification event. The 11q22 amplicons include, in addition to *YAP1*, several genes which could be considered as possible candidate oncogenes such as BIRC2 (cIAP1) and BIRC3 (cIAP2) encoding apoptosis inhibitor proteins. Evidences reported that BIRC2 cooperates with *YAP1* to accelerate tumorigenesis and promote mouse liver carcinogenesis [12]. In addition, several members of MMP gene family, known to be involved in extracellular matrix remodeling, in cell invasion and in metastasis development [53], have been proposed as oncogene targets of the 11q22 amplicon [54, 55]. Finally, progesterone receptor (PGR) is only 1 Mb proximal to *YAP1* and its co-amplification may play a role in female cancers. Therefore, the combined oncogenic role of co-amplified genes within 11q22 cannot be ruled out and remains a complex and fascinating issue to be explored.

Interestingly, we found that five of the significantly modulated genes identified as targets of *YAP1* are components of Hippo pathway (*STK3*/*Mst2*, *STK4*/*Mst1*, *LATS2, NF2* and *TEAD2*). Specifically, TEAD2, which is a transcription factor that interacts with *YAP1* to promote the expression of the Hippo responsive genes [56] results to be upregulated after *YAP1* silencing. All the other modulated genes represent negative growth regulators acting in a kinase cascade that ultimately inactivates *YAP1* and we revealed that they are downregulated upon *YAP1* silencing. Therefore, considering that Hippo pathway is a cascade of phosphorylation events which inhibits *YAP1*, we can speculate that in *YAP1*-amplified cancer cell lines the transient *YAP1* downregulation, by mimicking an over-activation of Hippo pathway, induces a feedback loop function on Hippo pathway components, that tends to counterbalance the *YAP1* silencing.

In conclusion, our results demonstrate that in the *YAP1*-amplified cancer cell lines under study the *YAP1* gene effectively sustains multiple transformed traits, indicating that *YAP1* is a direct oncogenic target of the 11q22 amplicons. In addition, we directly detected that *YAP1* amplification is actually present in a variable but significant fraction of carcinoma subtypes.

## MATERIALS AND METHODS

### Cancer cell lines

Ca-Ski and EKVX cell lines were purchased from ATCC, RO82 cell line from Interlab Cell Line Collection, Genova, Italy (ICLC). Ca-Ski and EKVX were grown in RPMI 1640 (Sigma) supplemented with 10% heat-inactivated FBS (Lonza), RO82 cell line was grown in DMEM (Sigma), Ham's F12 (Lonza), MCDB 105 (Sigma) (2:1:1) supplemented with 10% heat-inactivated FBS (Lonza), in a humidified incubator at 37°C and 5% CO_2_ (Thermo Electron Corporation). Cell identity was monitored by microsatellite typing and absence of mycoplasma contamination was checked regularly.

For stable *YAP1* silencing experiments, short-hairpin RNA (shRNA) expression vectors were used targeting *YAP1* (shY1 SH-012200-02-10, shY2 SH-012200-03-10, Thermo Scientific) or an off-target sequence (shOT, s-004000-02, Thermo Scientific) as negative control. Cells were infected with MOI 5-10 in the presence of Polybrene (4 ug/mL), cells were selected using puromycin 72 hours post infection and bulk cell population was collected and screened for *YAP1* expression.

For transient *YAP1* silencing, cells were transfected using the Dharmafect Transfection Reagent (Thermo Scientific) and small-interfering RNAs (siRNA) targeting *YAP1* (si*YAP1*) (AM16708A-107951, AM16708A-107952, AM16708A-114602, Ambion) and an off-target sequence as negative control (siOT, AM4611 Ambion).

In order to establish Ca-Ski xenograft, cells (10^6^) were injected subcutaneously into the each flank of six-week-old immunocompromised athymic nude mice (Harlan). Tumor size was monitored weekly. Tumor volume was calculated using the formula 1/2r^3^. Animal experimentation was approved by Institutional IRB and performed according to National laws. The data showed the means and the s.e.m of three independent experiments, each composed of 4 mice per experimental condition. Two classes' comparisons with multiple measurement points have been performed by two-way analysis of variance.

### Tumor samples

Paraffin sections of 21 human cervical cancer samples, 15 human thyroid cancer samples, 56 human CSN tumor samples and of 77 human non small cell lung cancer samples were obtained from the Departments of Pathology of collaborating centers (Treviso General Hospital, Italy; “Fondazione Pascale” of Naples, Italy; “Fondazione Istituto Nazionale Tumori” of Milan, Italy).

### Protein expression

For western blotting, 40 ug of protein lysates were used as described [57]. Antibodies used and conditions are listed in Supplemental Table 4. Protein expression was analyzed using Odyssey infrared imaging system (Li-Cor) and the data were normalized on siOT and on shOT. Immunohistochemistry was performed using anti-*YAP1* antibody diluted 1:25 (Cell Signaling, 4912) using Ultra vision detection system (LabVision), upon heat-induced epitope retrieval. Endogenous peroxidase was blocked with 0.3% hydrogen peroxide in methanol for 30 minutes. Intensity scoring of tumor cells was performed by pathologist based on a 4-tiered scale. The comparisons between two classes were performed by Fisher two tailed extract test by grouping negative/1+ cases and 2+/3+ cases.

### Anchorage-independent growth

Cells (10^5^) were resuspended in 0.35% agar complete medium and seeded on 0.5% bottom agar medium in 60 mm dish. After two weeks, plates were stained with iodonitrotetrazolium violet (1mg/ml, Sigma) and clones were counted at the microscope (Olympus). The data showed the means and the s.e.m of three independent experiments. The comparisons between two classes were performed by two sample unpaired t-test.

### Cell proliferation assay

Cell proliferation was assessed by cell counting using an automated cell counter (Bigital Bio) and by Sulforhodamine B (SRB) assay as described [58]. The comparisons between two classes were performed by two-sample unpaired t-test. Cisplatin (Teva) was added 24 hours post- plating at the indicated concentrations. The data showed the means and the s.e.m of three independent experiments. The comparisons between two classes were performed by two-sample paired t-test. The IC50 (concentration of drug inhibiting 50% of cell growth) was calculated applying non-linear fitting dose response.

### Annexin V evaluation

Cells (1×10^5^) were plated in 6-well plate and treated with cisplatin (Teva) 100 uM for 16 hours. The data were acquired and analyzed using Guava instrument (Millipore). The data showed the means and the s.e.m of three independent experiments. The comparisons between two classes were performed by two-sample paired t-test.

### Bromodeoxyuridine (BrdU) assay

BrdU Flow Kit (BD Pharmingen) was used to quantify cells that were actively synthesizing DNA. Cells were pulsed with 1 (Ca-Ski and RO82) or 2 (EKVX) hours of BrdU incorporation, washed and released for additional 1 hour in complete medium. Stained cells were measured and analyzed with flow cytometer (Beckman Coulter). The data showed the means and the s.e.m of three independent experiments. The comparisons between two classes were performed by two-sample paired t-test.

### Fluorescent in situ hybridization (FISH)

FISH analyses were performed on cells smeared over a positively charged slide and on human non small cell lung cancer samples that were arrayed on a tissue microarray. BAC DNA probes used for FISH were extracted using QIAprep Midiprep (Qiagen), verified by sequence tagged site content mapping and labeled with Spectrum Orange deoxyuridine triphosphate or Spectrum Green deoxyuridine triphosphate (Vysis, Downers Grove, IL) by use of a Nick Translation Kit (Vysis) according to the manufacturer's instructions. The FISH hybridization signals were analyzed in an Olympus BX51 microscope coupled to a charge-coupled device camera COHU 4912 (Olympus). The images captured were analyzed using the Mac Probe software (PowerGene Olympus). A minimum of 100 nuclei were counted.

### Real-time PCR

Total RNA was extracted using EZ1 RNA cell mini kit (Qiagen) on a Bio-Robot EZ1 (Qiagen) according to the manufacturer's instructions and 500 ng of RNA was retro-transcribed using Super Script III RT (Invitrogen) with random primers. Relative quantification of gene expression was performed in triplicate using TaqMan assays on Demand on a ABI Prism 7900HT Sequence Detection System (Applera) by comparative Ct method, using the HPRT gene (HPRT PDAR, 4326321E) (Applied Biosystem) as endogenous reference control and shOT and siOT cells as calibrators. The list of genes investigated and primer sets used is in Supplemental Table 4. Quantitative PCR was also used to determine *YAP1* DNA copy number status using relative quantification method with standard curves. The RNaseP gene (RNaseP, 4401631 Applied Biosystem) was used as a reference gene. Real time PCR data were analyzed by the SDS software 2.3, in order to obtain the Ct and the standard deviation. In each experiment, standard curves were analyzed for both reference gene (RNaseP) and target gene using serial diluitions of a normal control DNA. Using the standard curves of reference gene and target gene, the mean Ct of individual samples were converted to nanogram equivalent of template DNA. The obtained values were converted to target gene copy number by normalizing over a normal donor DNA as calibrator.

### Wound healing assay

Exponentially growing cells (8×10^5^) were plated in 60 mm dish. Cell monolayer was scratched using a pipette tip. Complete medium was added after washing in PBS, wound closure was monitored every 24 hours and pictures were taken by bright-field microscopy (Olympus).

### Time-lapse video microscopy

Cells (3×10^3^) were plated in 24-well plate. After overnight cell adhesion, cells were incubated at 37°C in 5% CO_2_ atmosphere in the Leica Time Lapse AF6000LX workstation equipped with the Leica DMI 6000 motorized microscope and an environmental chamber for the proper setting of temperature humidity and CO_2_ concentration. The allows the acquisition of the Images were collected every 5 minutes for 12 hours using AF6000 Software (Leica) and were analyzed with IM2000 software (Leica), to obtain the total distance covered (micron) by the cells. The data showed the means and the s.e.m of three independent experiments. The comparisons between two classes were performed by two-sample paired t-test.

### Chemotaxis experiments

Transwell permeable supports, 6.5 mm diameter inserts, 8.0 um pore size, polycarbonate membranes (Corning Inc.) were used to perform migration assay. Cells (10^5^) were seeded in the upper chamber of the transwell insert in serum-free medium (Sigma). The lower chamber of the transwell was filled with 600 uL of culture medium containing 10% of fetal bovine serum. Cells were incubated at 37°C and 5% CO_2_ for 16 h, then transwells were removed and stained with 0.1% Crystal Violet (Sigma) in 25% methanol. Non-migrated cells were scraped off the top of the transwell with a cotton swab. Migrated cells were quantified by eluting crystal violet with 1% SDS and reading the absorbance at 550 nm using the microplate reader Infinite 200 (TECAN). The data showed the means and the s.e.m from one representative experiment out of three. The comparisons between two classes were performed by two-sample paired t-test.

### Bioinformatic analyses

*YAP1* target genes in 11q22-amplified cancer cell lines were analyzed by gene expression profiling experiments. Class comparison analysis performed using the BRB Array Tool software from the US National Cancer Institute (linus.nci.nih.gov/BRBArrayTools.html) and comparing *YAP1* proficient cells versus *YAP1*-silenced cells. Differential expression levels of individual probe sets were considered statistically significant if p <0.001 by random variance t tests. A multivariate permutation test was applied to provide 90% confidence that the false discovery rate was less than 10%. Such stringent significance threshold was used to limit the number of false-positive findings. The statistically significantly modulated genes identified were functionally annotated using Ingenuity bioinformatic tool.

The analysis of *YAP1* copy number (deletion or amplification) status in independent datasets was performed using Oncomine (www.oncomine.org) and Sanger Centre (www.sanger.ac.uk/genetics/CGP/CellLines) database repositories, two cancer microarray databases which contain gene copy number data collected from the literature and proprietary datasets. The comparison between *YAP1* deletion and amplification was performed in order to test the null hypothesis that the two anomalies are expected at the same frequency by Chi-square test.

## SUPPLEMENTARY FIGURES AND TABLES




