# Supplementary material for: YAP1 acts as oncogenic target of 11q22 amplification in multiple cancer subtypes
Source: Oncotarget. 2014 Mar 23;5(9):2608–21. doi: 10.18632/oncotarget.1844 (PMC4058031; doi:10.18632/oncotarget.1844)
Supplement: Supplementary file 1 [file oncotarget-05-2608-s001.pdf]

***YAP1* acts as oncogenic target of 11q22 amplification in multiple cancer subtypes – Lorenzetto et al**

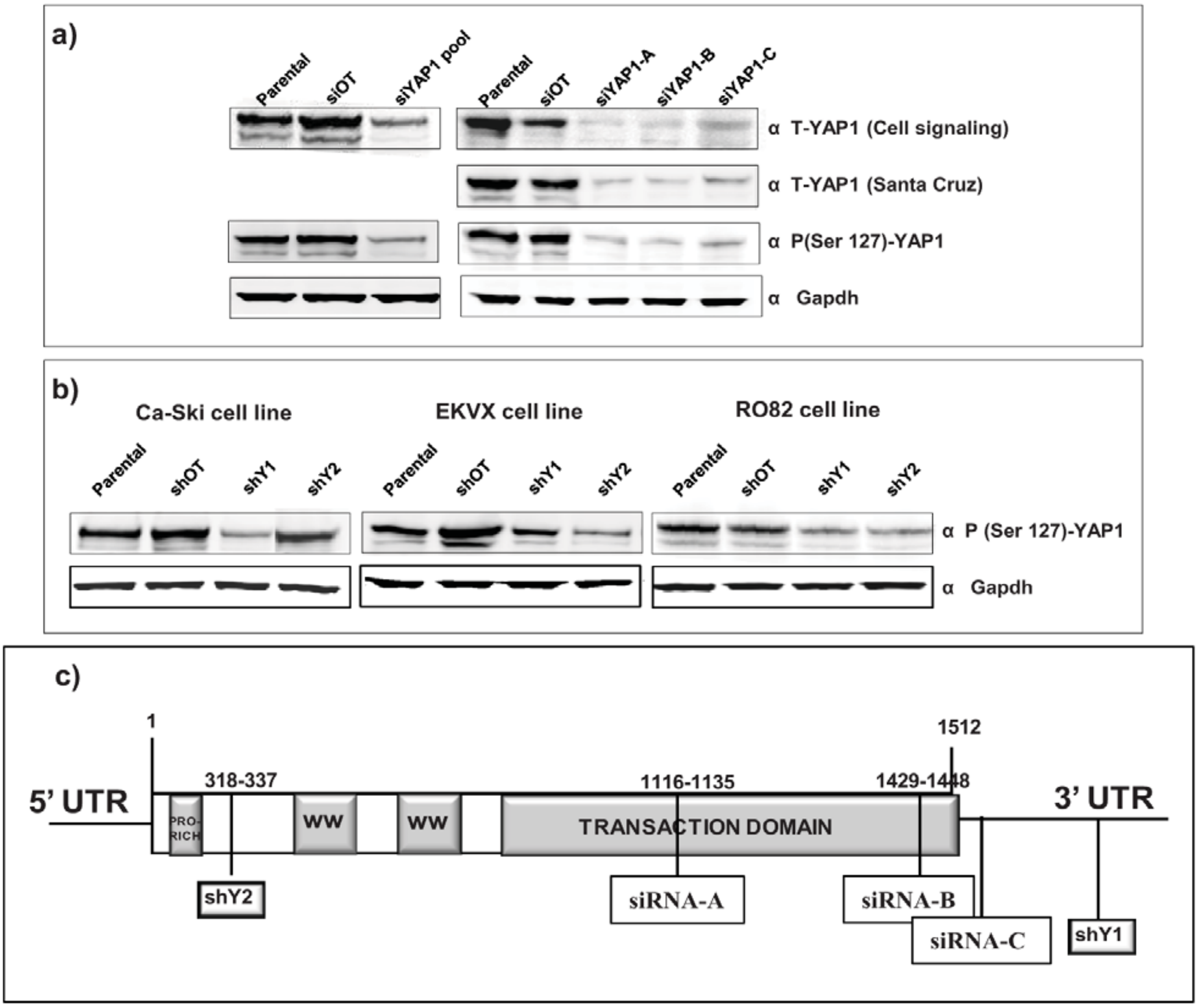

Supplemental Figure 1. Effective *YAP1* silencing in 11q22-amplified cancer cell lines. **a)** In EKVX cell line, total and phospho-*YAP1* protein levels of parental cells, off target cells (siOT) and of *YAP1* silenced cells (si*YAP1*) were determined by western blot analysis. On the left *YAP1* silencing effect of the pool of the three different siRNAs targeting *YAP1* and on right the *YAP1* silencing effect of the single constructs (si*YAP1*-A, si*YAP1*-B, si*YAP1*-C). The panels show the residual *YAP1* protein expression level detected using two different antibodies for total *YAP1* (Santa Cruz and Cell Signaling) and one antibody for the *YAP1* phosphorylated form at Ser127 relative to GAPDH. **b)** Ca-Ski, EKVX and RO82 protein bulk cell population after puromycin selection of parental cells, off target cells (shOT) and *YAP1* silenced cells (shY1 and shY2) was defined by western blot analysis for the phospho (Ser127)-*YAP1*. GAPDH was used as loading control. **c)** Location of siRNA and shRNA sequences targeting *YAP1*.

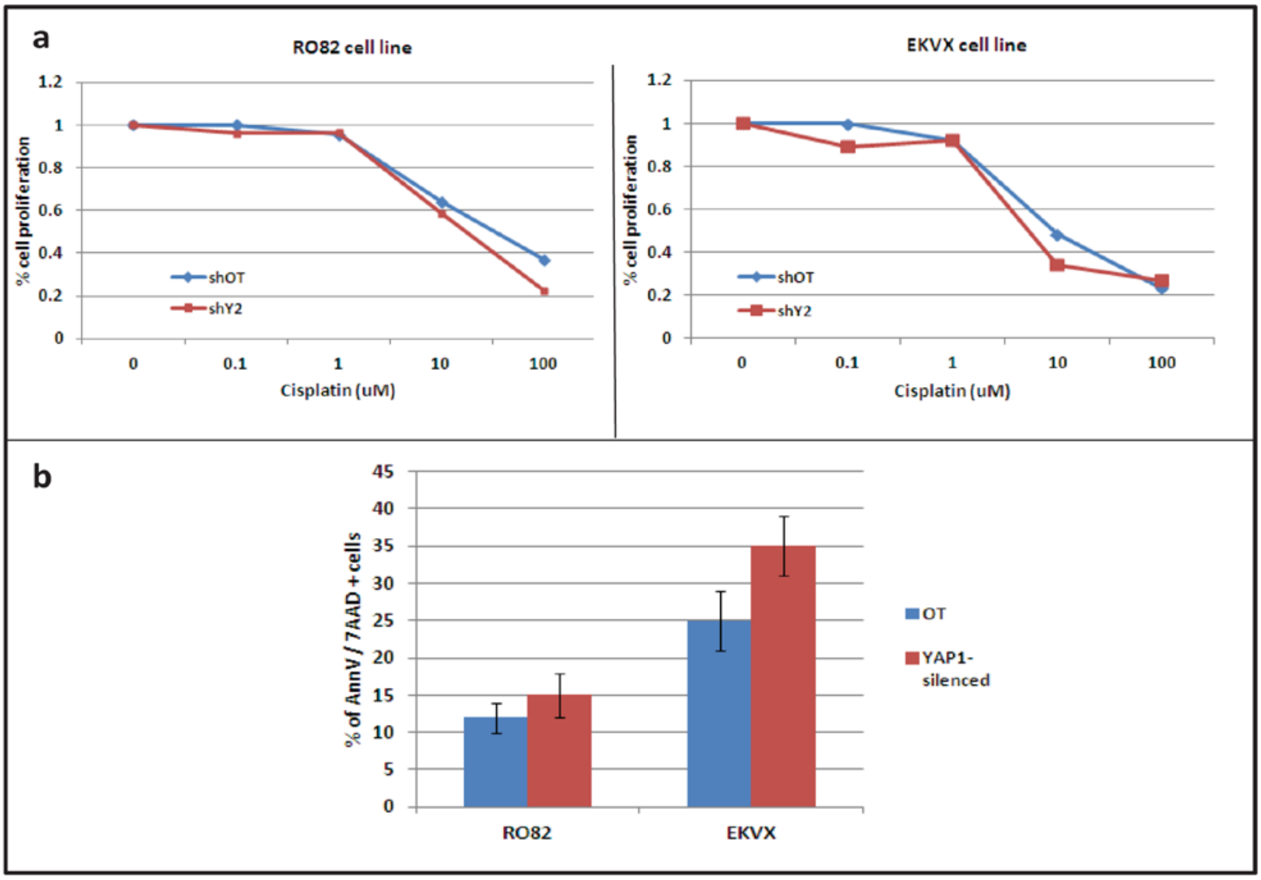

Supplemental Figure 2. *YAP1* silencing increases DNA damage response in RO82 and EKVX cell lines. a) Percentage of cell proliferation reduction determined by SRB assay in RO82 and EKVX off target and *YAP1*-silenced cells after cisplatin treatment. b) Flow cytometer analysis was performed on off target cells and *YAP1* silenced cells. The histograms showed the mean $\pm$ SEM percentage of single or double annexin V and 7AAD positivity cells after cisplatin treatment.
